# Swedish primary healthcare practitioners’ perspectives on the impact of arts on prescription for patients and the wider society: a qualitative interview study

**DOI:** 10.1186/s12913-021-07258-7

**Published:** 2021-11-26

**Authors:** Anita Jensen, Hilary Bungay

**Affiliations:** 1 Competence Center for Primary Healthcare, Clinical Research Center, Region Skåne, Sweden; 2 School of Allied and Public Health, Faculty of Health, Education, Medicine, Social Care, and Education, Anglia Ruskin University, Chelmsford, UK

**Keywords:** Arts on prescription (AoP), Arts on referral, Primary healthcare, Social prescribing, Mental health, Healthcare practitioners, Wellbeing

## Abstract

**Background:**

There is growing evidence that participating in arts activities are beneficial for mental health and wellbeing. Many patients attending primary care services have mental ill-health or social issues that healthcare practitioners currently do not have adequate ways of supporting. This study set out to explore the perspectives of primary healthcare practitioners on Arts on Prescription (AoP) as an additional referral pathway.

**Methods:**

A qualitative exploratory descriptive approach within an interpretive framework using semi-structured interviews was used to explore healthcare practitioners’ perspectives and experiences of AoP programmes in Sweden*.* Ten interviews were conducted with healthcare practitioners in primary care. Data were analysed using an inductive thematic approach.

**Results:**

The healthcare practitioners interviewed, recognised the need for more holistic approaches to care for those with mental health issues. They perceived that AoP is beneficial for patients in terms of motivation, creating routines, providing social interactions, and increasing self-esteem. In addition, AoP was felt to have the potential to impact upon current service provision and wider society. However, whilst the opportunity to refer patients to AoP in conjunction with conventional treatments was valued, participants reported that time pressures on practitioners and the continuing dominance of the medical model of care were barriers to wider acceptance amongst practitioners at the present time.

**Conclusions:**

AoP enabled primary healthcare practitioners to offer an additional pathway for patients that is an adjunct to the traditional care pathway. However, the programmes tend to be project-based and often time limited. For programmes to be sustainable and be included as part of a wider range of interventions available to healthcare practitioners’ suitable levels of funding would be required.

**Supplementary Information:**

The online version contains supplementary material available at 10.1186/s12913-021-07258-7.

## Background

Mental health is of global concern with close to a billion people living with a mental health disorder, and the COVID-19 pandemic has further exacerbated the problem [1]. In Sweden both mild-to-moderate and severe mental health disorders also represent a significant burden to individuals and society. Mild and moderate mental health problems amount to the highest number of presenting cases and these have been on the rise over past decades [2]. Patients attending with mental health problems are common in primary healthcare in Sweden [3] where mood and anxiety disorders, and stress and adjustment disorders account for a large proportion of the burden on services [4]. General practitioners (GPs) in Sweden estimated that approximately one third of patients in primary care have problems that could be attributed to psychosocial causes [5]. Whilst this is relatively old data similar findings are seen in the UK where an estimated 15% of primary care patients consult the GP for non-medical problems [6]. Although there are no more recent studies from Sweden, like many other countries Sweden is faced with increasing problems related to mental ill health [7] and according to the Public Health Agency of Sweden 20% of the population have been diagnosed with depression at some point in their lives [8]. Furthermore, findings from a recent study suggest that because of the COVID-19 pandemic, levels of depression and anxiety have increased significantly with the strongest predictors of these levels being poor self-rated overall health and a history of mental health problems [9]. The use of arts activities for mental health and wellbeing is now commonplace in some parts of the world [10, 11]. Within primary health care provision, such activities are frequently offered through social referral programmes and often referred to as AoP [12]. This is a form of social prescribing that enables GPs, nurses, and other primary care staff to refer patients to a range of local and non-clinical services [13]. In a UK study, Healthcare practitioners (HCPs) and health workers who referred patients to an AoP programme considered it to be therapeutic, relaxing, providing a safe environment, social benefits and greater options for health workers when helping patients with complex social problems. AoP programmes were clearly valued by referrers [14]. Exploring barriers and enablers to social prescribing for patients with mental health problems, a study with GPs in the UK found that there is a need for a more systematic feedback structure, more formal training about social prescribing and developing the relevant inter-personal skills [15].

Although AoP programmes are relatively new in Sweden [16], physical activity have been prescribed to patients by practitioners in primary health care for almost two decades [17]. Many practitioners also have the option of offering nature rehabilitation and mindfulness as part of the palette of non-clinical activities available to support patients’ physical and mental health wellbeing. There is an increased understanding that individual health is determined by a range of social economic, and environmental factors described as the social determinants of health [18, 19], and social prescribing offers a holistic approach to addressing individuals’ needs. One of the aims of social prescribing (and therefore AoP) is to reduce pressures on primary healthcare [20] and signpost patients towards services that can support their needs where pharmaceutical approaches may fail or might in themselves be considered inadequate. This study aims to explore the experiences and perspectives of Swedish primary healthcare practitioners who have referred patients to AoP.

## Method

### Research design

A qualitative exploratory descriptive approach within an interpretive framework using semi-structured interviews was used to explore healthcare practitioners’ perspectives and experiences of AoP programmes in Sweden. In this article the research design and data are reported in accordance with the COREQ guidelines [21].

AoP programmes in Sweden were identified that employ facilitators that are artists (not therapists) to deliver AoP programmes for adults (aged over 18 years) experiencing mental health issues (not dementia). Programmes were included that were free for the participants to attend, provided a range of visual or performance based participatory arts activities in community settings for a minimum of six weeks at least one time per week and had a referral process in place. Only four AoP programmes met these criteria and therefore a pragmatic approach to sample size was adopted to capture the range of knowledge and experiences of all the relevant programmes. In addition, only practitioners that had referred to AoP in the past two years were invited to participate in the study. The AoP programmes vary in structure and delivery depending on collaborations, resources, and location in Sweden, but are all aimed at people experiencing mental health issues such as stress, anxiety and mild to moderate depression and those who are socially isolated.

Purposive sampling was used to ensure only healthcare practitioners who had referred patients to an AoP programme and therefore had knowledge and experience of the programmes were included in the study [22]. Inclusion criteria for practitioners included referral of one or more patients to a programme, and the ability to provide consent. The AoP programme managers were contacted via email and phone and asked to support recruitment of potential HCPs to the research and some HCPs were contacted directly by the researcher via email. The programme manager made initial contact with the HCP to gauge their interest in participation in the study. Once this has been confirmed, the researchers contacted the healthcare practitioners to provide further information about the study. Some participants were also contacted directly. In Sweden, primary medical centres are staffed by a range of HCPs and those approached to participate in the study included GPs, nurses, mental health workers, health counsellors, psychologists, physiotherapists, and psychotherapists. Therefore, the views of different HCPs were captured.

### Data collection

To answer the research aims of the study participants were asked about their experiences of referring patients to a programme, reasons for referral, understanding of AoP, impact of AoP, and the financial implications of referring to such programmes (see supplementary file 1 interview guide). The interview guide was sent to the participants prior to the interview so they were able to consider their responses in preparation for the interview. The interviews were semi-structured and were all conducted in English via zoom or in person, they ranged in length from 21 to 52 min with the median of 45 min and all gave written, informed consent. All 10 interviews were conducted by the first author who was known by two of the participants. To minimise the risk of potential bias the second author XX was blinded to participants’ identity on the interview transcripts and data analysis was initially conducted separately and concurrently. The protocol development and data analysis were undertaken both authors. XX (she/her) is a researcher in Arts & Health who has untaken qualitative research with patients, service users, healthcare staff and cultural workers in a range of different settings and countries over the past 8 years. XX (she/her) is a Professor of Arts, Health and Wellbeing and has undertaken qualitative research with patients, service users and staff across a range of health and community settings over the last 20 years. The interviews were audio-recorded and transcribed verbatim for analysis. Transcripts were anonymised using pseudonyms and checked for accuracy against the original recording.

### Data analysis

Data analysis followed an interpretive and inductive approach, with the data explored to ensure the analysis reflected the accounts of the participants and responded to new themes as identified. To ensure accuracy and dependability of the data analysis XX checked the transcripts against the audio recordings. This also familiarised the XX with the data (Step 1 of Braun and Clarke’s stages of analysis) [23]. Thematic analysis was conducted systematically and followed Braun and Clarke’s six stages of analysis (Step 1: Become familiar with the data, Step 2: Generate initial codes, Step 3: Search for themes, Step 4: Review themes, Step 5: Define themes, Step 6: Write-up) [23]. To ensure rigour and trustworthiness both authors independently read each interview transcript and made notes of potential codes, and through discussion a list of codes was created (step 2). The codes were then applied across the data set with detailed coding undertaken in discussion with both authors. The coded data was then searched for patterns to develop sub-themes which were then clustered and organised into the final themes by XX and XX (steps 3–5). Table 1 provides an overview of the themes and Table 2 provides an example of the coding process, and the development of a sub-theme and theme. The participants code index can be understood as: SE = Sweden, 1 = participant no., HCP = Healthcare practitioner.

**Table 1:**
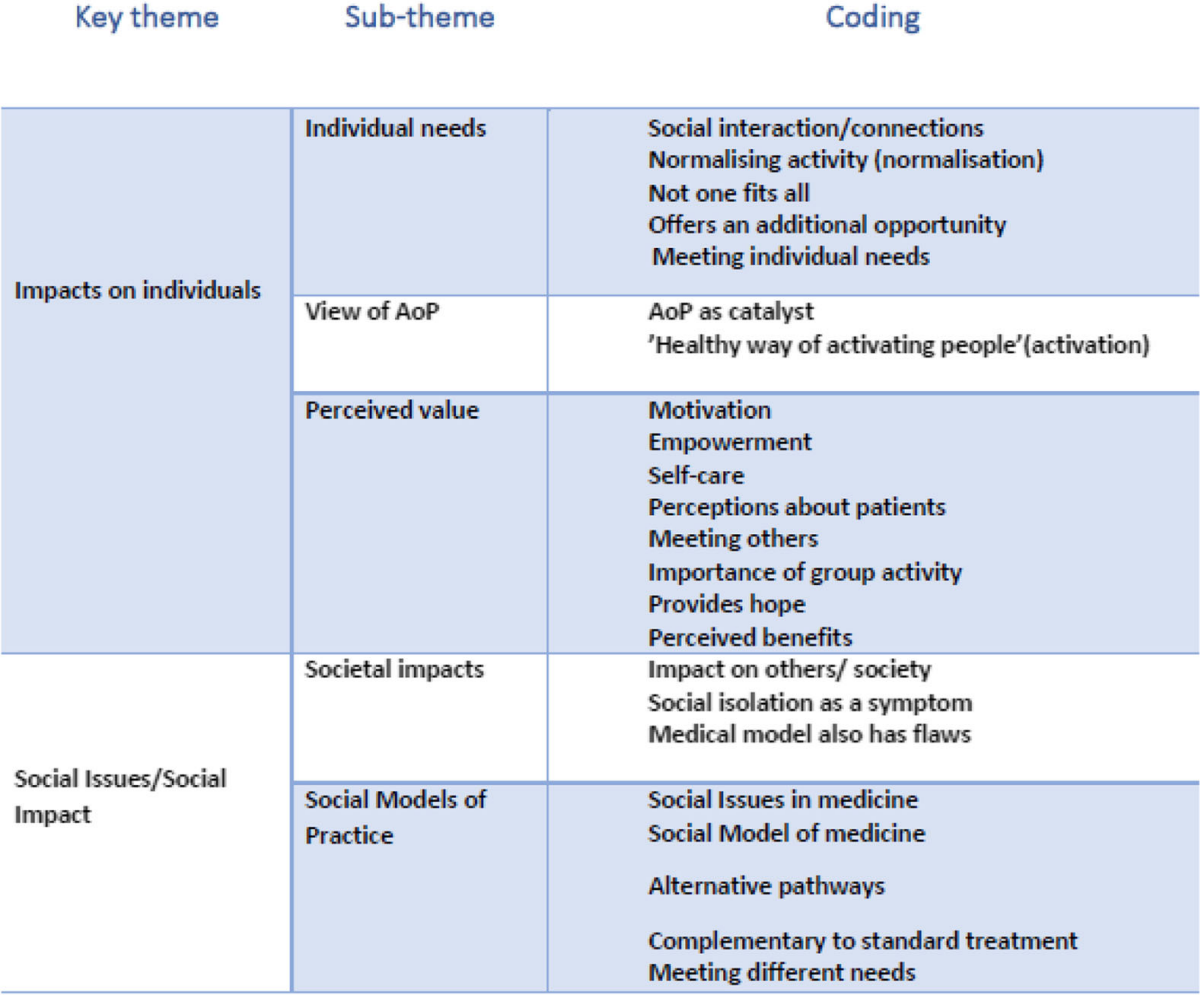
Themes

**Table 2:**
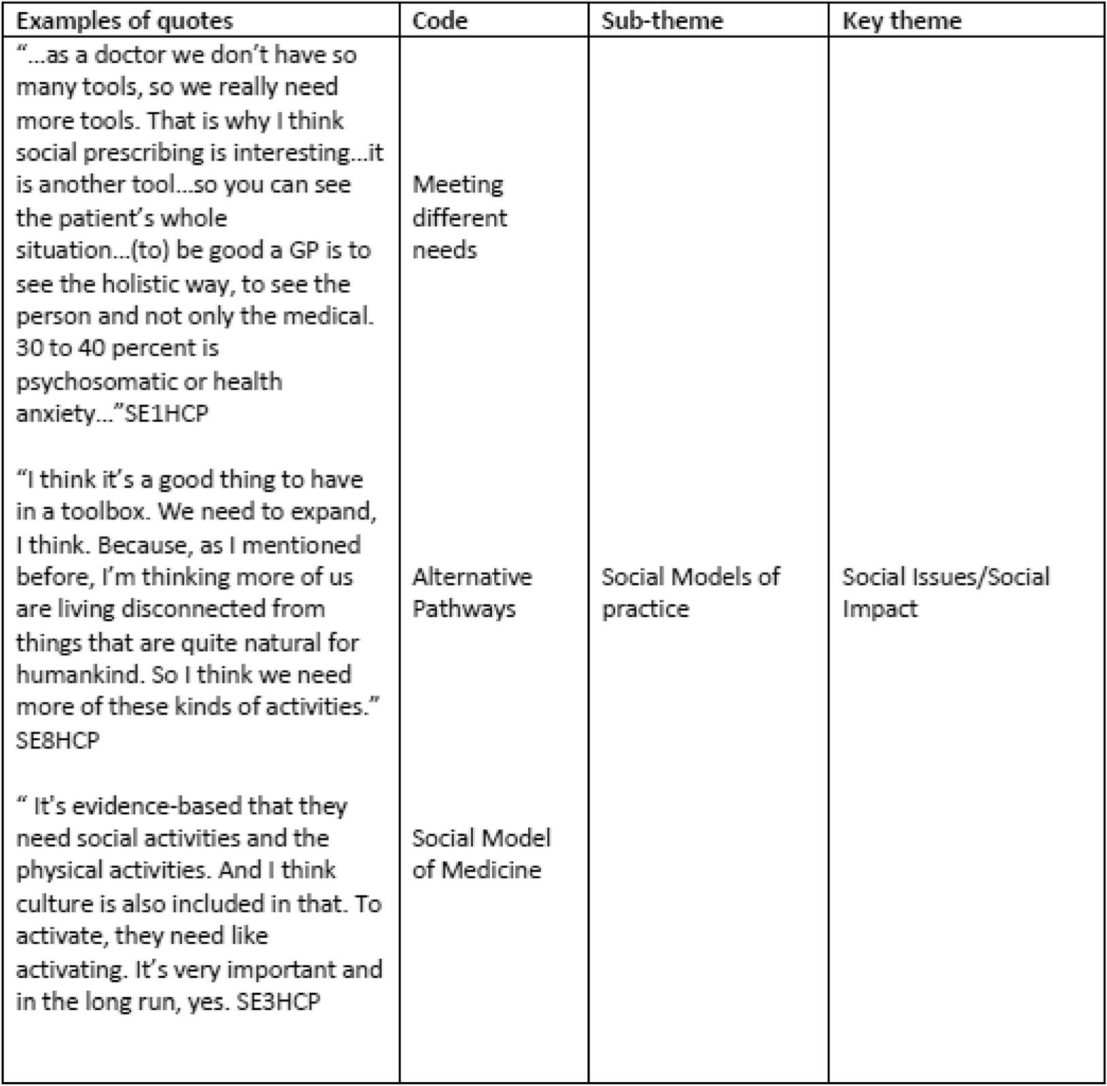
Coding process and theme development

## Findings

Data analysis identified two key themes. The first theme, *Impacts on Individuals* encompasses the HCPs’ beliefs about the programme and the perceived impact on the patients, including social interactions, motivation, a normalising activity and meeting individual needs. The second *Social Issues and Social Impact* relates to the impact of the programmes and includes the need for alternative pathways to meet individual needs, the medical model of care, and the issue of social isolation. The analysis of findings is presented as a narrative with quotes from the different HCPs’ used to illustrate the points made.

### Impacts on individuals

Across the dataset, it became evident that all referrers believed that participation in AoP programmes had a positive impact on patients, providing a routine, opportunities for meeting others, widening social networks, and were motivating. It was also noted that it was thought that AoP enables individual needs to be accommodated, an important element of patient-centred care. In this way, the programmes were perceived to be of particular value through offering patients ways to increase their levels of self-confidence and self-esteem by extending networks and increasing social interactions:

“The patients mentioned hope and motivation, strengths, like, self-awareness, self-esteem and that kind of thing. Social interactions and culture”. SE3HCP

“I think it's very important that they [patients] come out, can interact with other people in other activities than those they are used to. It gives them [patients] greater self-esteem and confidence in doing new things”. SE2HCP

Positive changes were observed in patients who had completed an AoP programme. With HCP noting changes in the patients’ demeanour during the practitioner-patient interaction:

“But, all in all, I think it’s only positive. And that’s not only their reflection, it’s also something I can see in the room when I meet patients afterwards, where I work with them. This [AoP] is something that has helped my patients”. SE8HCP

However, another HCP gave the example of a patient with social anxiety who was referred to a programme. The patient had attended most of the sessions and had provided positive feedback about the activities, enjoying being part of the group. Despite this, the patient had reported to the HCP how the prospect of going back to work still provoked anxiety. This might indicate that AoP was perceived to provide a ‘safe space’ but also demonstrates a limitation of a time limited programme. The process of recovering from mental health problems and reconnecting with the community needs to be approached with a long-term view. Therefore, whilst AoP may act as a catalyst for moving on to other things there is also the need for other initiatives to follow up or to be offered simultaneously depending on the individual patient:

“ … I think ten weeks is not enough … you have to do something more because this is a very long process. If you have been ‘out from life’ for maybe two or three years you can’t get it back in ten weeks. This is a starter I think, it could be a way to start to get motivated to do something else”. SE1HCP

All HCPs interviewed said that it is important to be able to offer different pathways to individual patients. This emerged as an emphasis on person-centred care and highlighted an understanding that patients are individuals with personal needs and preferences. The option of being able to offer a range of different activities was compared to having different medicines to offer:

“Yes, a palette … so they can choose because people are individuals, and they are different and it's the same for the doctor prescribing medicine. You cannot give the same medicine for depression to everyone. It's like you must think of who you meet and what they need … ” SE4HCP

It was also acknowledged that AoP may only appeal to some patients, but one participant remarked however, that they had changed their point of view regarding this following referring patients to programmes:

“When I first heard of it, I thought no one would like it but I was wrong. I thought the group of people that would like this, was a few people who were already interested in art, and especially I thought part of the group would say, “I don't want to dance, no, I don't like to perform.” But I was wrong. So wrong”. SE9HCP

HCPs reported that patients said that gaining a routine by attending the programmes was of value as was being able to practice social interactions. Furthermore, being in a group with other people who are referred with similar health issues was also seen as positive element of the programme. A patient had commented that they felt to be part of a group of people, with similar problems had helped to normalise his/her mental health problems. Similarly, it was stated that feedback from patients included:

“They've been mentioning, "Well, now, I have routines." It's probably the first thing they say. "It's so nice to have a routine. I know that that day, I need to leave home." It sounds like nothing for somebody who doesn't have depression, of course, but if you have anxiety or have depression, it's really worthwhile that you have that in your schedule. They also said;” It’s so nice to see that I'm not the only one. It's so nice that I've been able to open up a little bit." People with social anxiety say, "Now, I have a platform for exposure training." So, there are so many gains from being in a group”. SE4HCP

There were also comments on the need for patients to be “activated” and that was important in the long term. Such activation would have psychosocial benefits and was recognised as an alternative to counselling or to be offered alongside therapeutic initiative:

“I think more psychosocial activities are needed instead because we do have [mental health] counsellors”. SE7HCP

“It's evidence-based that they [patients] need social activities and physical activities. And I think culture is also included in that. To activate, they [patients] need activation. It’s very important in the long run, yes”. SE3HCP

Whilst the above explores the perceived impacts on individual patients in the following section the wider perceived impacts are explored under the theme *Social Issues* and *Social Impact.*

### Social Issues and Social Impact

*Social Issues* and *Social Impact* is a broad theme and included HCPs reflecting on the challenges of current service provision, the need for alternative forms of provision, and difficulties in overcoming the dominance of the medical model of care. Considering the impact of AoP, some HCPs discussed issues around the challenges of needing more options or tools to provide satisfactory care and accommodate the needs of their patients. Where ‘tools’ refer to different interventions and services, it was perceived that there was a shortfall in healthcare provision to address the growing numbers in the population presenting with mental health issues. For example, one HCP commented on current options as not being adequate or addressing the problem:

“There are so many people with mental health problems. What should we do?” We can see that what we’re doing [traditional interventions] is not enough because it doesn’t really help”. SE7HCP

Not being able to provide adequate care and lacking opportunities to support patients with mental health problems was associated with wider societal issues creating challenges in primary healthcare and causes frustrations for HCPs. A need for alternative provision for people with mental health issues to cater for the social causes of ill-health was identified. The lack of different options and services to refer patients to or provide opportunities for those patients beyond conventional treatments was seen as a limitation of current provision:

“Sometimes, we're locked here. We don't have that many possibilities to activate the patient, actually, outside this room”. SE4HCP

It was also recognised that whilst patients seek care for physical health problems, in reality they were looking for care and support for the social issues in their lives, resonating with the existing literature:

“There are a lot of studies already showing that quite a lot of somatic patients seeking somatic care, but it is actually social health that they are looking for”. SE8HCP

Welcoming new pathways such as social prescribing and a need for holistic approaches to health care was a common thread across the data. The option of providing activities for people to work with animals and engaging with nature were suggested as ways of expanding the current healthcare toolbox and AoP was considered a helpful programme to have in the toolbox when considering an expansion in provision in primary care:

“ … as a doctor we don’t have so many tools, so we really need more tools. That is why I think social prescribing is interesting … it is another tool … so you can see the patient’s whole situation … (to) be good a GP is to see the holistic way, to see the person and not only the medical. 30 to 40 percent is psychosomatic or health anxiety … “SE1HCP

This was supported by another HPC who considered having alternative provisions as an essential component of healthcare.

“It's like an integral way of seeing healthcare. It's not rocket science, actually. It's like having several legs on a chair; it provides more stability...” SE4HCP

Although AoP was perceived as an activity that could add to the services available in primary care, it was also mentioned that the Swedish Social Insurance Agency was a community stakeholder that valued the AoP programme, because of the benefits for people on long term sick leave.

Participatory activities such as AoP provide HCPs with more options to offer patients and fill some of the gaps in current provision as well as creating alternative pathways for patients in primary health who are not responding to current treatments or indeed require other directions. However, a recurrent issue identified by participants was the dominance of the medical model of care in practice and the impact this had on current attitudes and referral practices. The dominance of the medical model of care when dealing with patients with mental health problems was highlighted by a HCP who reflected on how it was still common to prescribe antidepressant medication despite evidence suggesting that people experiencing mild to moderate depression would benefit from alternative interventions such as physical activation. It was, however, also acknowledged that a shift away from the medical model and treatments to alternative approaches would require time:

“The doctors still prescribe antidepressant medicine when we know that the effect of physical activation is actually more effective, not when it comes to severe depression, but mild to moderate depression. But still, it takes time”. SE4HCP

These observation about activation resonate with previous research that found that patient activation is about patient’s confidence, motivation, and the ability to manage their health.

Prescribing medicine was perceived as habitual, with practitioners adopting the pharmaceutical route because they are used to prescribing medicine for physical illnesses with little consideration for other ways of dealing with certain mental health conditions. This was believed to be exacerbated by the existing pressures and stressors in primary care:

“ … they forget that there are also other things to prescribe … I think maybe it’s lack of time or lack of, yes, I think it’s a lot of lack of time, actually. It’s so much stress in these clinics nowadays, so they just do the most prioritised thing”. SE6HCP

Prescribed medicines in themselves may treat the symptoms but cannot address the social factors that impact upon mental health. The HCPs interviewed identified that patients with mental health issues also experience social isolation and loneliness. This was described as an increasing problem and concerns were expressed about how the Covid-19 pandemic was adding to the number of patients experiencing social isolation and loneliness. It was noted the impact of this on primary care provision and that it may become more significant in the future:

Lifestyle issues and loneliness issues, I think that's going to be more important in the long term because I think the problems that we must deal with are social issues, social problems. I think those things we should work more with in primary care than we do now”. SE3HCP

Issues presented in primary health care services have the potential to impact on society more widely. Therefore, being able to provide adequate primary healthcare care which addresses the social determinants of health not only impacts positively on the individual but also on communities and society.

Already in Sweden, primary healthcare offers services such as mindfulness sessions, courses in handling stress, yoga, and physical activity on prescription. AoP is an additional opportunity, and its impact was discussed in the context of current provision. Being able to offer AoP was seen as beneficial for both the patient and the HCP and in turn for society. It was seen as especially beneficial for patients on long term sick leave and revolving door patients:

“I think the impact on the wider perspective, I think it’s a win-win situation really. It’s not only beneficial for the patients, it’s also beneficial for the healthcare settings … our patients they come every second month with the same problems, and they come back, and they come back, and they come back. But if this will be beneficial, it will be a win-win situation for both patients and society really”. SE6HCP

“It’s another way to help these people who are very, very far from the work market and who are feeling very bad. You don’t have any tools to help them because you have tried everything before, and this is a new way and maybe an injection for them to do something to help them … ” SE1HCP

In summary, AoP is perceived to have a beneficial impact on the individual patient but is also understood to have an impact on current service provision and society. AoP offers an additional activity to offer patients alongside existing other more conventional treatments.

## Discussion

A major strength of this research is that it is the first study to explore the perceptions of healthcare practitioners’ perceptions and experiences of referring patients with mental health issues to AoP programmes in Sweden. Those interviewed had referred patients to one of the four programmes included in the study, providing an overview of the current context for AoP in this setting.

It was found that the HCPS perceived direct impacts on individual patients in terms of increased confidence, and motivation and that patients valued the opportunity for group interaction, meeting others with similar conditions and experiences. References were also made to the health and wellbeing benefits of activation for patients. This concurs with existing literature from Denmark and the United Kingdom which reports how participating in AoP programmes results in increased self-esteem, self-confidence, and motivation, to develop social relations and connections the community, and be to a catalyst to move on in life [24, 25]. AoP is a person-centred approach, however, as the findings from this study suggest it is not only perceived to be beneficial for the individual but has a wider scope and as other studies suggest the potential to reduce pressures on primary healthcare services [26]. In addition, a recent survey of GPs in the UK found that 72% out of 1002 of respondents supported the notion that arts-based interventions and activities can make a significant contribution to improving the health and wellbeing of the NHS workforce [27].

The interviewees in this current study highlighted the need for alternative pathways for HCPs to offer patients presenting with mental health issues. It was recognised that social factors impact on patient’s health and wellbeing, but addressing the underlying problems and concerns of the patients might not be within the capacity of primary healthcare providers [28]. Many patients’ concerns and mental health problems emerge from their social situation, and the management of long-term medical conditions is often influenced by individual circumstances [29]. Participants also acknowledged that patients may not differentiate between medical and non-medical problems when attending primary care, since the impact of these are often interwoven [6, 30]. Consequently, HCPs can feel overwhelmed and frustrated at not being able to offer adequate care and solutions as the interviewees in this study suggest. This supports findings from an earlier Swedish study which reported that one third of GPs (*n* = 191) considered that their current knowledge and experience were insufficient to provide adequate help to patients with psychosocial problems [5].

There is a growing demand from policy makers and healthcare practitioners for more wide-ranging sources of holistic support, including non-medical care, which has prompted the emergence of social prescribing initiatives in diverse international contexts. In New Zealand, Canada, Holland, and Japan and in the UK, we see examples of social prescribing being embedded in the health service [31, 32]. The Scandinavian countries have similar models of healthcare yet there is currently a lack of relevant research from Sweden. However, a Danish study (*n* = 1500) showed that patients that who booked a GP consultation with one main reason/problem often had one or more additional problem categorised as psychological or social. Furthermore, 23% of patients had additional psychological problems and 16% of patients had additional social problems [33]. Such numbers emphasise a growing problem for GPs dealing with mental health and social problems, and as the findings in this study have identified, feel that they lack the tools to deal with these issues.

In the UK social prescribing is a key component of ‘Universal Personalised Care’ of the National Healthcare System (NHS) due to be unfurled by 2023/24 [34]. Social prescribing link workers will connect people to wider community support helping them to improve their health and wellbeing and to address some of the underlying causes of ill-health. The social determinates for health influence health outcomes and can been understood as the conditions in which people are born, grow, live, work, and age, and the wider circumstances and systems shaping daily life [18]. Countless people are challenged by circumstances that are beyond their control (financial trouble, unsafe neighbourhoods, or discrimination and so on). Relationships and interactions with family, friends, colleagues, and community members can also have a significant (negative) impact on health and wellbeing during a lifetime [18, 19].

Whilst social prescribing cannot address all the social determinants of health or social inequalities, a holistic and personalised way addressing symptoms and problems through non-medical support could complement conventional health treatments. However, participants identified several barriers to HCPs considering alternative care pathways for patients presenting with mental ill- health. Firstly, there remains a tendency to revolve around the dominant medical model of practice and it was suggested that some HCPs might be locked into this way of thinking, and secondly, due to the time pressures they are not able to consider other interventions. This is in line with a Swedish study showing that two thirds of GPs surveyed did not think they had sufficient consultation time for patients with psychosocial problems [5]. Likewise, a Danish study found that GPs rarely stated social factors as being related to the presented problem and generally paid more attention to the biomedical aspects of ill-health when dealing with psychological issues [33]. This is an issue for wider society as overprescribing of medicines has substantial costs associated with it, both socioeconomically and to those receiving the medication [24]. Common problems are given a diagnosis, stigma increases, and people are often left with no resources to be able to adjust to or understand their problems this can have significant adverse consequences that often mostly affect the most vulnerable groups of patients.

A major issue that was raised in this study and needs to be considered more widely is the need for sustainability of provision and long-term approaches to promoting mental health. HCPs in this current study experienced the introduction of initiatives which are often short-term and considered more of a burden than a positive development due to the existing pressures to in the healthcare sector. This requires that appropriate structures are embedded into primary healthcare services rather than the short-term projects which are often introduced as short-term fixes.

## Limitations

It is acknowledged that there are some limitations to this study. The sample size is limited because of the small numbers of HCPs who currently refer to the few AoP programmes in Sweden. However, as such it provides an understanding of HCPs perspectives of the programmes and will help to inform the implementation of future programmes. The interviews were semi-structured and conducted in English so both researchers could access the data. For some participants this meant that they were being interviewed in their second language. To mitigate for this all interviewees were sent a copy of the interview questions prior to the interview so they had time to consider the answers beforehand. A further limitation in understanding healthcare practitioners’ perceptions and knowledge of AoP programmes was that only people who had referred to the programme were interviewed, and their responses were all very positive. It would have been interesting to interview practitioners who had not referred to the available programmes to explore the reasons for non-referral and the information about the programmes that would make AoP an attractive additional option.

## Conclusion

This paper presents the findings from an exploratory study looking at HCPs’ perceptions of referring patients with mental health issues to AoP programmes. AoP has only relativity recently been introduced in Sweden and there is a paucity of research investigating the introduction and use of the existing programmes. In addition, as discussed in the introduction, whilst research has been conducted looking at the impact of AoP on participants, there is a lack of research examining healthcare practitioners’ perspectives of the impact such programmes. This study indicates that those primary HCPs interviewed recognised the need for a more holistic approach to care for those with mental health issues. However, in common with the UK, AoP programmes are project-based rather than funded as a mainstream service and consequently they are often time limited, which means awareness of the programmes is difficult to maintain. For programmes to be sustainable and be included as part of the palette of activities and interventions available healthcare practitioners’ suitable levels of funding would be required.

## Supplementary Information

**Additional file 1.**

## Data Availability

All data generated or analysed during this study are included in this published article [and its supplementary information files].
