# Supplementary material for: Swedish primary healthcare practitioners’ perspectives on the impact of arts on prescription for patients and the wider society: a qualitative interview study
Source: BMC Health Serv Res. 2021 Nov 26;21:1277. doi: 10.1186/s12913-021-07258-7 (PMC8626132; doi:10.1186/s12913-021-07258-7)
Supplement: Supplementary file 1 — Additional file 1. [file 12913_2021_7258_MOESM1_ESM.pdf]

Supplementary file 1: Interview guide
Health practitioners' perceptions of Arts on Prescription

1. **Background information**
    - How long have you worked as [Health Practitioner] in this area?
    - Location of service (Rural/Urban, Size of population served, Socio-economic character of the area?)

2. **Reasons for referral**
    - Where/when did you first hear about [name of organisation]?
    - Do you feel sufficiently informed about the programme? If yes, how do you get this information? If not, why not?
    - How many patients have you referred to the programme?
    - What are the most common conditions/symptoms patients you refer to the programme have?
    - What are your reasons for referring these patients to the programme?
    - Would you like to be able refer more patients to such programmes? If yes, why? If no, why not?
    - Is there any follow-ups with the patient after he/she has completed the programme? If yes, how? If no, why not?
    - What feedback have you had from the people you referred to Arts on Prescription?
    - Are there any changes you have noticed in the patients referred? (For example changes in attendance pattern, self-reported changes in wellbeing)

3. **Attitudes to Arts on Prescription**
    - What are your thoughts about a programme like arts on prescription?
    - How do you think Arts on Prescription fits within Social Prescribing and wider health/social provision?
    - To what extent do you think social interventions such as Arts on Prescription can contribute to individual health/wellbeing?

4. **Impact of Art on Prescription**
    - What impact do you think Arts on Prescription has on the individual and/or their family?
    - How does the availability of Arts on Prescription have an impact on the GP practice/primary care service?
    - What if any impact does Arts on Prescription have on the wider community?

5. **Financial implications**
    - Does your service pay towards the Arts on Prescription programme? If yes how much/ if no why not – what would you think a programme of interventions costs?
    - Are you aware of how [name of organisation] funded?

6. To what extent does your service collaborate with the Arts on Prescription programme organisers?
7. What opportunities would you like to collaborate with the programme organisers?
8. There is a growing evidence base supporting the use of arts for health and wellbeing- what is your experience/knowledge of research in this area?
